# A Simple and Fast Non-Radioactive Bridging Immunoassay for Insulin Autoantibodies

**DOI:** 10.1371/journal.pone.0069021

**Published:** 2013-07-29

**Authors:** Ingrid Kikkas, Roberto Mallone, Nadia Tubiana-Rufi, Didier Chevenne, Jean Claude Carel, Christophe Créminon, Hervé Volland, Christian Boitard, Nathalie Morel

**Affiliations:** 1 Commisariat à l'Energie Atomique, iBiTec-S, Service de Pharmacologie et d'Immunoanalyse, Commisariat à l'Energie Atomique Saclay, Gif sur Yvette, France; 2 Institut National de la Santé et de la Recherche Médicale, U1016, Cochin Institute, DeAR Lab Avenir, Paris, France; 3 Paris Descartes University, Sorbonne Paris Cité, Faculté de Médecine, Paris, France; 4 Assistance Publique Hôpitaux de Paris, Hôtel Dieu, Service de Diabétologie, Paris, France; 5 University Paris, Hôpital Robert Debré, Pediatric Endocrinology, Paris, France; Centro di Riferimento Oncologico, IRCCS National Cancer Institute, Italy

## Abstract

Type 1 diabetes (T1D) is an autoimmune disease which results from the destruction of pancreatic beta cells. Autoantibodies directed against islet antigens are valuable diagnostic tools. Insulin autoantibodies (IAAs) are usually the first to appear and also the most difficult to detect amongst the four major islet autoantibodies. A non-radioactive IAA bridging ELISA was developed to this end. In this assay, one site of the IAAs from serum samples is bound to a hapten-labeled insulin (GC300-insulin), which is subsequently captured on anti-GC300 antibody-coated 96-well plates. The other site of the IAAs is bound to biotinylated insulin, allowing the complex to be detected by an enzyme-streptavidin conjugate. In the present study, 50 serum samples from patients with newly diagnosed T1D and 100 control sera from non-diabetic individuals were analyzed with our new assay and the results were correlated with an IAA radioimmunoassay (RIA). Using IAA bridging ELISA, IAAs were detected in 32 out of 50 T1D children, whereas with IAA RIA, 41 out of 50 children with newly diagnosed T1D were scored as positive. In conclusion, the IAA bridging ELISA could serve as an attractive approach for rapid and automated detection of IAAs in T1D patients for diagnostic purposes.

## Introduction

Type 1 diabetes (T1D) is an autoimmune disease characterized by the destruction of insulin-producing pancreatic beta cells within the islets of Langerhans. During this autoimmune process, autoantibodies are generated that react against several beta-cell antigens, e.g. insulin, glutamic acid decarboxylase (GAD65), protein tyrosine phosphatase (IA-2) and zinc transporter 8 (ZnT8). These autoantibodies can be present years before disease onset [1], allowing for an early diagnosis before clinical manifestations. Moreover, measuring these autoantibodies allows etiologic diagnosis of a given diabetes case and adaption of treatment accordingly.

Insulin autoantibodies (IAAs) are usually the first to appear before T1D development and they are most frequently found in young children, as their level and prevalence at diagnosis inversely correlate with age [2]. One of the current methods for the detection of T1D autoantibodies is enzyme-linked immunosorbent assay (ELISA), in which the immobilized antigen captures autoantibodies from the sample and detection is achieved using labeled antigen [2], [3]. However, this method cannot be applicable when measuring IAAs, because it appears that human IAAs cannot react with insulin directly bound to plates [4], [5]. IAAs are usually measured by radioimmunoassay (RIA), which is based on immunoprecipitation of ^125^I-labeled insulin. However, RIA is expensive, requires newly synthesized radiolabeled antigen for each set of assays, takes more than 24 h to carry out and requires handling and disposal of radioactive products. Recent studies have used electrochemiluminescence (ECL) detection developed by Meso Scale Discovery (MSD) as a method for measuring IAAs [5], [6]. Although this technique does not require synthesis of radiolabeled antigens, dedicated equipment is needed, with a relatively high cost compared with most other technologies. Poor correlation between laboratories taking part in international workshops has repeatedly been reported with RIA, with an average low sensitivity for IAA detection [2], [7], [8]. Clearly, there is a compelling need for new and better methods to measure IAAs in terms of sensitivity, cost and time requirements.

We describe the development of a non-radioactive bridging IAA assay, where bivalent IAAs are bound to two insulin moieties in solution, thus forming a bridge. This liquid-phase technique allows most insulin epitopes to be available for binding, which is not the case when insulin is directly bound to plates. For the present study, 50 serum samples from patients with newly diagnosed T1D and 100 control sera from non-diabetic individuals were analyzed. The performance of our IAA bridging ELISA was compared with that of an IAA radioimmunoassay kit (RSR Limited Cardiff, UK) validated by the Diabetes Antibody Standardization Program (DASP). In addition, the sensitivity of our ELISA was compared with that of an electrochemiluminescence assay performed with the MSD technology under the same conditions.

## Materials and Methods

### Serum Samples

50 serum samples from newly diagnosed T1D children (26 males, 24 females; mean age 8.8 years; range 0–18) and 100 control sera from non-diabetic individuals (65 males, 35 females; mean age 7.9 years; range 0–18) were analyzed. All samples were obtained before the start of exogenous insulin therapy. Local ethics committees approved the study.

### Assay Reagents and Equipment

Biotinamidohexanoic acid N-hydroxysuccinimide ester (NHS-LC-biotin) and recombinant human insulin expressed in yeast were from Sigma-Aldrich. A mouse monoclonal anti-insulin antibody (IN-05) was from Antibodies-online GmbH (Atlanta, USA). The production and selection of monoclonal anti-microcystin MC159 used for this study were described previously [9]. Sulfo-TAG N-hydroxysuccinimide [NHS]-ester was from MSD. When performing immunoassays, all reagents were diluted in enzyme immunoassay (EIA) buffer, i.e. 0.1 M phosphate buffer pH 7.4 containing 0.15 M NaCl, 0.1% bovine serum albumin (BSA) and 0.01% sodium azide. Plates were washed with washing buffer (0.01 M phosphate buffer pH 7.4 containing 0.05% Tween 20).

Immunometric assays were performed using Titertek microtitration equipment from Labsystem (Helsinki, Finland), including an automatic plate washer (Washer 120) and automatic plate reader (Multiskan Bichromatic). Microtiter 96-well plates (Maxisorp) were from Nunc (Roskilde, Denmark).

### Labeling of Insulin with Biotin

Biotin was covalently linked to insulin in a molar ratio 3∶1 and 10∶1 by reaction of an activated N-hydroxysuccinimide ester of biotin with the primary amino groups of the protein. The activated ester was dissolved in dimethylformamide (DMF) and added to a 0.1 M (pH 9.0) borax buffer solution of the protein (less than 5% final DMF concentration). After 30-minute incubation at room temperature, 100 µL of 1 M Tris-HCl buffer (pH 8.0) was added for 15 minutes, before completing with 500 µL of EIA buffer.

### Labeling of Insulin with GC300

A microcystin-LR analogue *N*-Boc-Adda [(2*S*,3*S*,8*S*,9*S*,4*E*,6*E*)-3-amino-9-methoxy-2,6,8-trimethyl-10-phenyldecadienoic acid] named GC300 was synthesized as previously described [10]. Ester-active GC300 was conjugated to insulin via its NH_2_ function. Briefly, 330 µg of insulin was dissolved in 165 µL of 0.1 M (pH 9.0) borax buffer. A three-fold molar excess (100 µg) of GC300 in DMF was added (6% final DMF concentration) to the insulin solution for 2 h at room temperature. The outcome of the coupling reaction was verified by mass spectrometry.

### HPLC Purification of Biotinylated and GC300-labeled Insulin

After coupling of insulin with biotin and GC300, the products were purified using the HPLC system ÄKTA purifier (GE Healthcare, Piscataway, NJ, USA) with a Chromolith performance RP 18E column (100–4.6 mm; Merck Chemicals). The mobile phase consisted of 0.1% aqueous formic acid: eluent A and 0.1% formic acid in acetonitrile: eluent B. The formic acid–acetonitrile gradient was run from 10 to 50% acetonitrile for 12.5- column volumes with a flow rate of 0.3 mL/min. Each sample (300 µg of either biotinylated or GC300-labeled insulin) was resuspended in 550 µL of 10% acetonitrile and 500 µL of this solution was injected. Elution was monitored at 280 and 220 nm. Fractions were collected, dried using a rotating vacuum concentrator (Eppendorf) and dissolved in 200 µL of EIA buffer. To identify the different coupling ratios of biotinylated insulin separated by HPLC, the collected fractions were characterized with a Voyager-DE STR MALDI-TOF mass spectrometer.

### IAA Bridging ELISA

**Figure 1:**
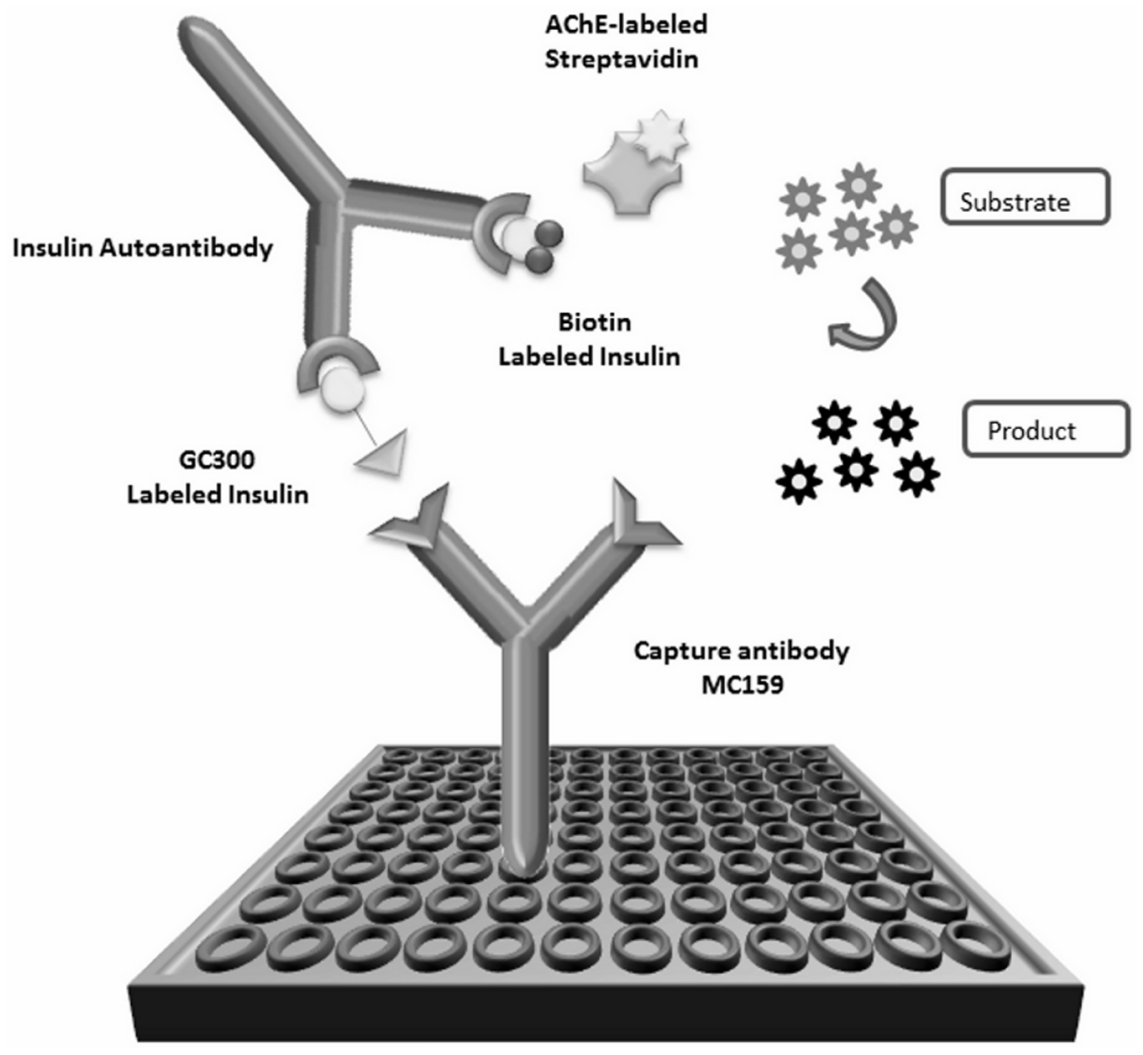
Principle of the IAA bridging ELISA. Serum sample or calibrator is incubated with GC300- and biotin-labeled insulin prior to transfer into anti-GC300 (MC159) mAb-coated wells. Serum IAAs form a bridge between GC300- and biotin-labeled insulin and this complex is captured on the solid phase of the MC159-coated plate. Biotin-labeled insulin bound to IAAs is detected using AChE-labeled streptavidin and the enzymatic activity is measured at 414 nm.

A series of experiments were carried out varying a range of assay parameters in order to establish the optimal conditions. Different concentrations of GC300-labeled insulin and biotin-labeled insulin were tested, ranging from 10 to 500 ng/mL. Furthermore, different incubation times (1, 2, 4 or 16 h) and temperatures with different serum volumes and dilutions were compared. Additionally, tests were made to verify the effect of human serum on the binding of insulin antibodies. Since acetylcholinesterase (AChE)-labeled streptavidin was used as enzymatic tracer [11], AChE activity was measured by the method of Ellman et al. [12]. Ellman's medium comprises a mixture of 7.5×10^−4^ M acetylthiocholine iodide (enzyme substrate) and 2.5×10^−4^ M 5,5′- dithiobis(2-nitrobenzoic acid) (DTNB) (reagent for thiol colorimetric measurement) in 0.1 M phosphate buffer (pH 7.4). Enzymatic activity was expressed in Ellman Units (EUs). One EU is defined as the amount of enzyme producing an absorbance increase of one unit during 1 min in 1 mL of medium, for an optical path length of 1 cm; it corresponds to about 8 ng of enzyme. The final assay set up is illustrated in Figure 1. Briefly, 96-well microtiter plates were coated with 100 µL/well of anti-GC300 monoclonal antibody (mAb) MC159 (10 µg/mL) in 50 mM phosphate buffer (pH 7.4). After 18 h of incubation at 20°C, plates were washed and blocked with 0.1% BSA-phosphate-buffered saline (PBS) for 24 h at 4°C. Serum samples (25 µL) were mixed with an equal volume of a 1∶1 mixture of biotinylated and GC300-labeled insulin (final concentration 200 ng/mL/each) in EIA buffer. After incubating for 1 h at room temperature, this solution was transferred into microtiter plates coated with MC159 mAb and reacted for 2 h at room temperature on an orbital shaker. The plates were subsequently washed 3 times and 100 μL of AChE-labeled streptavidin (2 EU/mL) was added to each well. After 1 h at room temperature followed by 3 washes, 200 μL of Ellman's medium was added to each well for 4 h. The absorbance was measured at 414 nm. As an internal positive control, a mouse anti-insulin mAb (IN-05) was used.

### Competitive Inhibition

Assessment of the competitive inhibition of IAA binding in nine IAA-positive serum samples and a control IAA-negative one was carried out by adding unlabeled insulin during the first incubation step with biotinylated and GC300-labeled insulin. Plate-bound IAAs were subsequently detected with the same ELISA format as described above.

### RIA

All serum samples were further analyzed by an IAA radioimmunoassay kit (RSR, Cardiff, UK), using the supplier’s protocol. Briefly, 20 µL of sera was reacted with 25 µL of ^125^I-(A14)-monoiodinated insulin (20,000 cpm) overnight at room temperature. The next day, 100 µL of anti-human IgG was added to each tube to precipitate any labeled insulin-antibody complex that had formed. After 1 h at 2–8°C, each tube was washed twice with 2 mL of cold assay buffer (50 mmol/L K_2_HPO_4_/KH_2_PO_4_ pH 7.0, 1% Tween 20, 0.5 g/L BSA, 0.5 g/L NaN_3_) and centrifuged at 1500×g for 20 minutes at 4°C. ^125^I-labeled precipitates were counted for 1 minute on a gamma counter, knowing that the amount of radioactivity in the precipitate was proportional to the concentration of IAA in the test sample. Positive and negative control sera and a set of assay calibrators containing different concentrations of insulin mAb were included in each assay. IAA titers above 0.4 U/mL (which corresponds to 2, 2% of bound ^125^I-insulin) were considered positive.

### Electrochemiluminescence Assay

High Bind Sector Imager 2400 96-well plates (MSD) were coated with MC159 mAb (40 µg/mL) in 50 mM phosphate buffer (pH 7.4). After 18-h reaction at 4°C, plates were washed and blocked with 5% BSA-PBS for 3 h. Four IAA-negative serum samples or PBS (25 µL) was spiked with anti-insulin mAb and mixed with 25 µL of GC300-labeled and ruthenium-labeled insulin (prepared with MSD Sulfo-TAG NHS-ester according to MSD instructions) in EIA buffer at a concentration of 200 ng/mL. After 1 h at room temperature, this solution was transferred into MSD plates coated with MC159 mAb and incubated for 2 h at room temperature on an orbital plate shaker. Plates were washed 3 times with washing buffer, 150 µL/well of 1×Read buffer (MSD) was added and plates were read with an MSD Sector Imager 2400 reader.

### Detection Limit of ELISA and ECL Assay

The five-parameter logistic fit (5-PL) function (GraphPad Prism 5) was used to model the characteristic curve for the IAA bridging ELISA and the ECL assay. The limit of detection for both assays was calculated by interpolating the average background signal plus 3 standard deviations on the standard curve.

## Results and Discussion

### Assay Optimization

Different assay formats have a significant impact on the optimal parameters used to maximize assay performance [13]. Anti-GAD and anti-IA-2 bridging ELISA tests have recently been developed that have outperformed classic liquid-phase RIA formats [3]. We tested several formats to find one suitable for IAA detection, and liquid-phase ELISA, using a bridging format, was found to be the most appropriate. As mentioned above, it appears that human IAAs cannot react with insulin directly bound to plates. Indeed, we observed no signal when directly coating insulin on the solid phase (data not shown). At least two different haptens are required for the liquid-phase ELISA format. In the current assay, IAAs form a bridge in solution between biotinylated insulin on one antigen-binding domain of IAAs and GC300-labeled insulin on the other. The GC300 hapten allows this complex to be captured on MC159 mAb-coated plates, whereas the biotin allows the complex to be detected by the streptavidin-conjugated tracer (Fig. 1). A GC300 molecule was chosen in this assay as it is a synthetic hapten which is not naturally found in the human body and for which a high-affinity mAb (MC159) is available to us. Insulin was labeled with both haptens at different hapten-to-antigen ratios and purified by HPLC. Using mass spectrometry, three different conjugates were observed for biotinylated insulin, namely insulin coupled with one, two or three biotin molecules. The most abundant conjugate was insulin coupled to 2 biotin molecules (45% of the total product). When performing assays with these different insulin-biotin conjugates, a 40% higher signal was obtained when using insulin coupled to 2 or 3 biotin molecules in comparison with insulin coupled to only 1 biotin molecule (data not shown). Using purified double-biotinylated insulin resulted in a higher signal (∼40%) than using biotinylated insulin before HPLC purification. This could be explained by the fact that HPLC purification eliminates unbound insulin (15% of the total product), which could otherwise bind to IAAs and lower the signal. For this reason, insulin coupled to 2 biotin molecules was used for subsequent assays. Other optimization steps included finding the most favorable concentrations of reagents (biotinylated and GC300-labeled insulin) for the assay. Tested concentrations ranged from 10 to 500 ng/mL and the balance for formation of bridging complex was achieved using 200 ng/mL of each reagent. The optimized parameters selected for subsequent experiments are described in *Materials and Methods*. The intra-assay coefficient of variation was 6.2% (n  = 8) and the inter-assay coefficient of variation was 5.8% (n  = 5) using an anti-insulin antibody-positive sample.

In most studies published to date using different non-radioactive IAA assays, sera are incubated with labeled haptens at 4°C for at least 16 h [5], [14], [15]. Remarkably, in the present IAA bridging ELISA, the signal did not change when incubating sera of T1D children at 4°C overnight or at room temperature for 3 h. Besides testing different concentrations and temperatures, different serum volumes and dilutions (from 4/5 to 1/10 dilutions) were compared and the optimal conditions were found to be 25 µL of serum diluted ½ in EIA buffer. Therefore, only 25 µL of the original serum samples is needed for this assay. Diluted sera were incubated with hapten-labeled reagents for 1 h at room temperature, followed by 2-h incubation on the mAb-coated capture plates.

**Figure 2:**
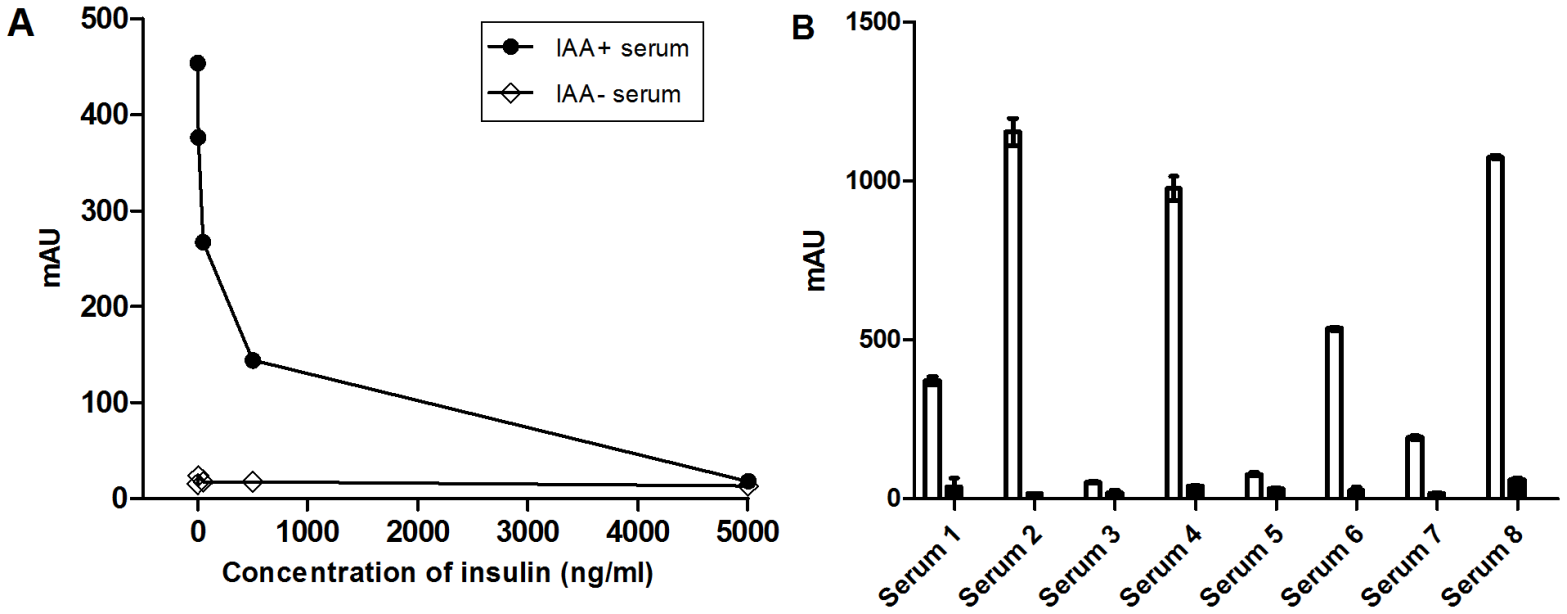
Competitive inhibition of IAA binding with unlabeled insulin. One IAA-positive and one IAA-negative serum sample (A) and eight IAA-positive serum samples (B) were incubated with 0, 5, 50, 500 and 5,000 ng/mL (A) or with 5,000 ng/mL (B) of unlabeled insulin together with biotinylated and GC300-labeled insulin in the first incubation step of the IAA bridging ELISA. White bars represent signals without addition of unlabeled insulin and black bars represent signals with addition of unlabeled insulin. mAU, milli-absorbance unit.

In order to ascertain the specificity of the assay, competition experiments were performed. An IAA-positive and an IAA-negative serum sample were incubated with serial dilutions of unlabeled insulin (0, 5, 50 and 5,000 ng/mL) together with biotinylated and GC300-labeled insulin (200 ng/mL/each; Figure 2A). In addition, eight serum samples with diverse titers of IAA were also incubated with 5,000 ng/mL of unlabeled insulin in the first incubation step of the IAA bridging ELISA (Figure 2B). As expected, addition of 5,000 ng/mL of unlabeled insulin completely inhibited IAA detection in the positive sera; however, the signal of the negative serum sample remained unaffected, indicating that the binding of IAAs to the capture mAb-coated surface is specific (Figure 2).

### Comparison of the Bridging ELISA with an ECL Assay

**Figure 3:**
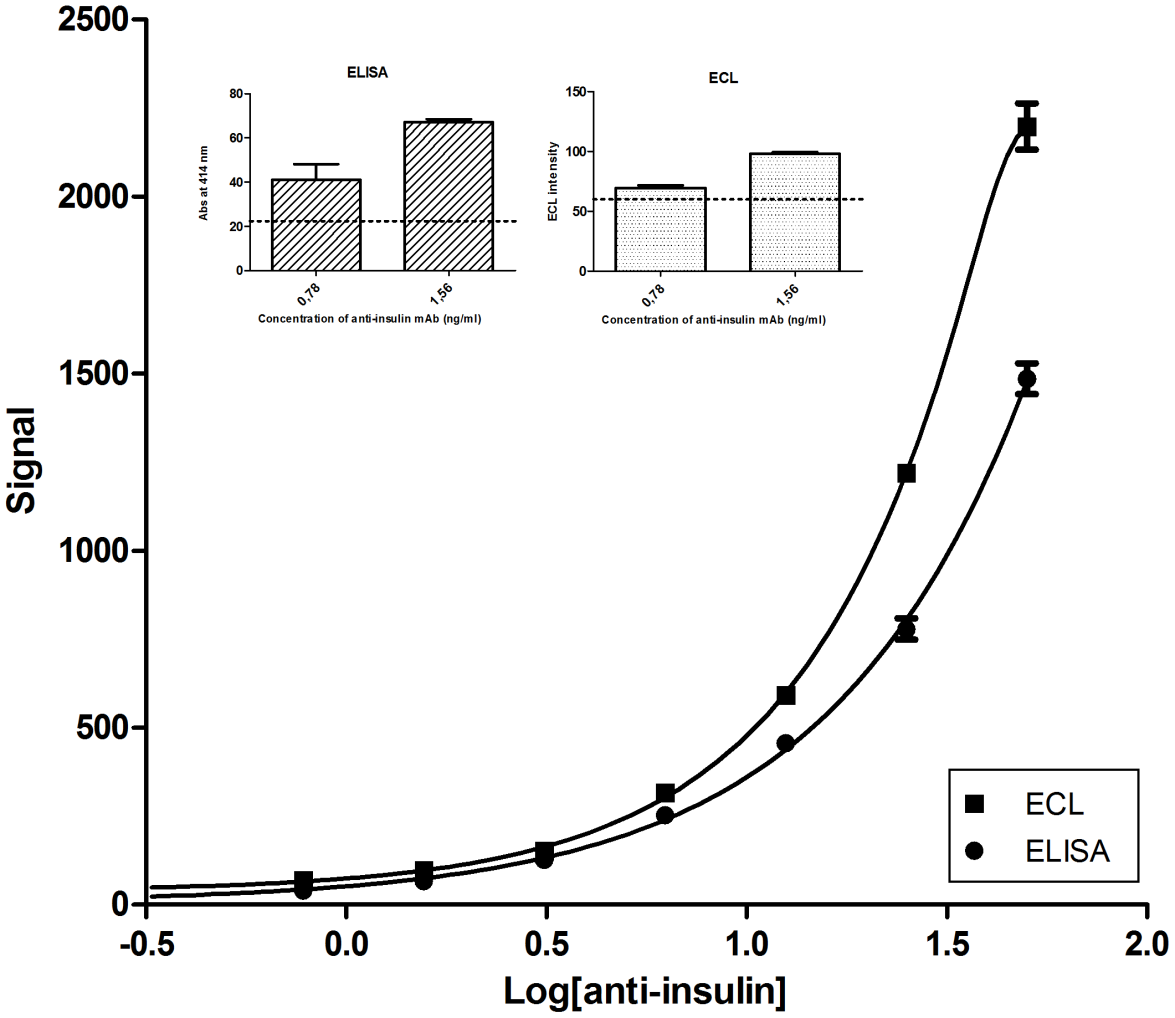
Comparison of the bridging ELISA and the ECL assay for the detection of anti-insulin mAb-spiked sera. An IAA-negative serum was spiked with serial dilutions of an anti-insulin mAb (0–50 ng/mL) and assayed in parallel with the two techniques with a 3-h incubation time. The five-parameter logistic fit was used to model the characteristic curve for both IAA bridging ELISA and ECL assay. The inset shows the low-concentration part of the curve and dotted lines indicate the positive cut-off values of the assays. Signal units: milli-absorbance units (mAU) for ELISA and ECL intensity for ECL.

Several studies have been published comparing ELISAs with ECL assays. ECL assays are reported to be 3 times [16] and up to 8 times [17] more sensitive than ELISAs. The sensitivity of bridging ELISA and the ECL assay was therefore compared, both techniques being performed using the same capture mAb and GC300 hapten coupled to insulin. Another insulin molecule was coupled to biotin for bridging ELISA, while for the ECL assay insulin was coupled to ruthenium. Both ELISA and ECL were separately optimized in terms of concentration of capture mAb, but the same incubation times, reagent concentrations and temperatures were used for both assays (see *Materials and Methods*). The analysis was done by comparing dilution curves of an IAA-negative serum spiked with anti-insulin mAb. When using the five-parameter logistic fit to model the characteristic curve for both bridging ELISA and ECL assay, it was found that the limit of detection was very similar for both techniques (0.6–0.85 ng/mL) (Figure 3).

**Figure 4:**
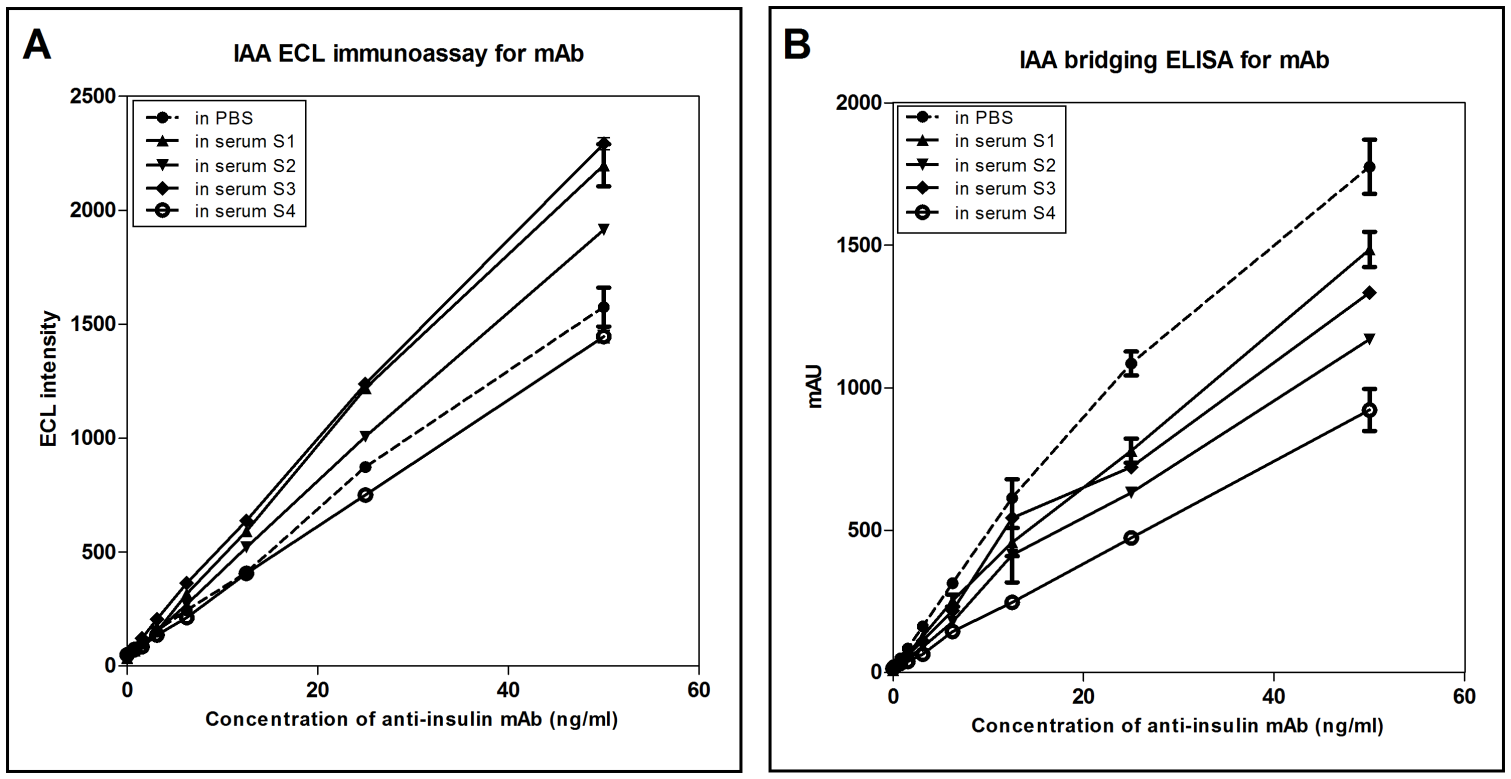
Binding of an anti-insulin mAb diluted in human serum versus PBS. Four IAA-negative human sera or PBS was spiked with anti-insulin mAb (0–50 ng/mL) and assayed using both IAA bridging ELISA (A) and IAA ECL assay (B). mAU, milli-absorbance units.

A study has been recently published using the MSD technology to assay IAAs from human serum samples. It was found that binding of insulin antibodies, either a mouse anti-insulin mAb or serum IAAs, was inhibited by normal human sera. To decrease this inhibition, a step of acid treatment of sera was introduced [5]. Interestingly, this phenomenon was only slightly observed when performing the MSD IAA assay using our bridging format. As shown in Figure 4A, no significant inhibition of binding of anti-insulin mAb was observed in normal human serum when assaying low concentrations of anti-insulin mAb compared with PBS. Similarly, no significant inhibition of anti-insulin mAb binding was observed in normal human serum compared with PBS for our IAA bridging ELISA when assaying low concentrations of anti-insulin mAb (Figure 4B). These results indicate that the human serum samples can be directly assayed with our ELISA method, thus eliminating a time-consuming pretreatment step.

### Assay Sensitivity and Specificity

**Figure 5:**
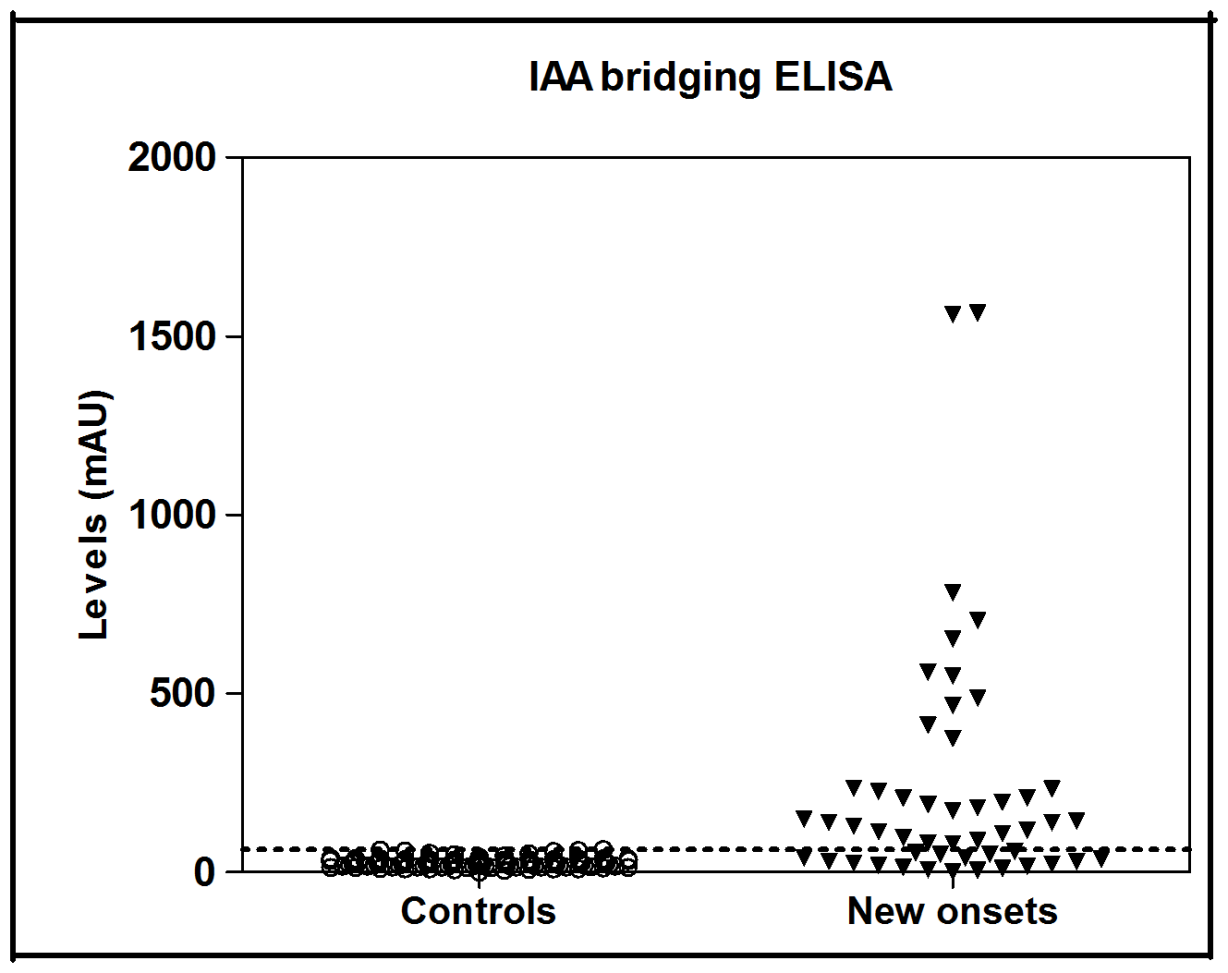
Analysis of serum IAAs using the IAA bridging ELISA. Sera were from 50 newly diagnosed T1D children and 100 age-matched control children. The dotted line indicates the cut-off value (64 mAU) based on the mean plus 3 SD of the control samples. mAU, milli-absorbance unit.

In order to validate our IAA bridging ELISA, IAA levels were assayed in serum samples from new-onset T1D (n = 50) and healthy control children (n = 100). The cut-off value of the IAA bridging ELISA was determined based on the mean plus 3 standard deviations (SD) of the control samples and which corresponded to 64 mAU (milli-absorbance units). Using this cut-off, IAAs were detected in 32 out of 50 (64%) T1D children and in 0 out of 100 (0%) healthy controls (Figure 5).

**Figure 6:**
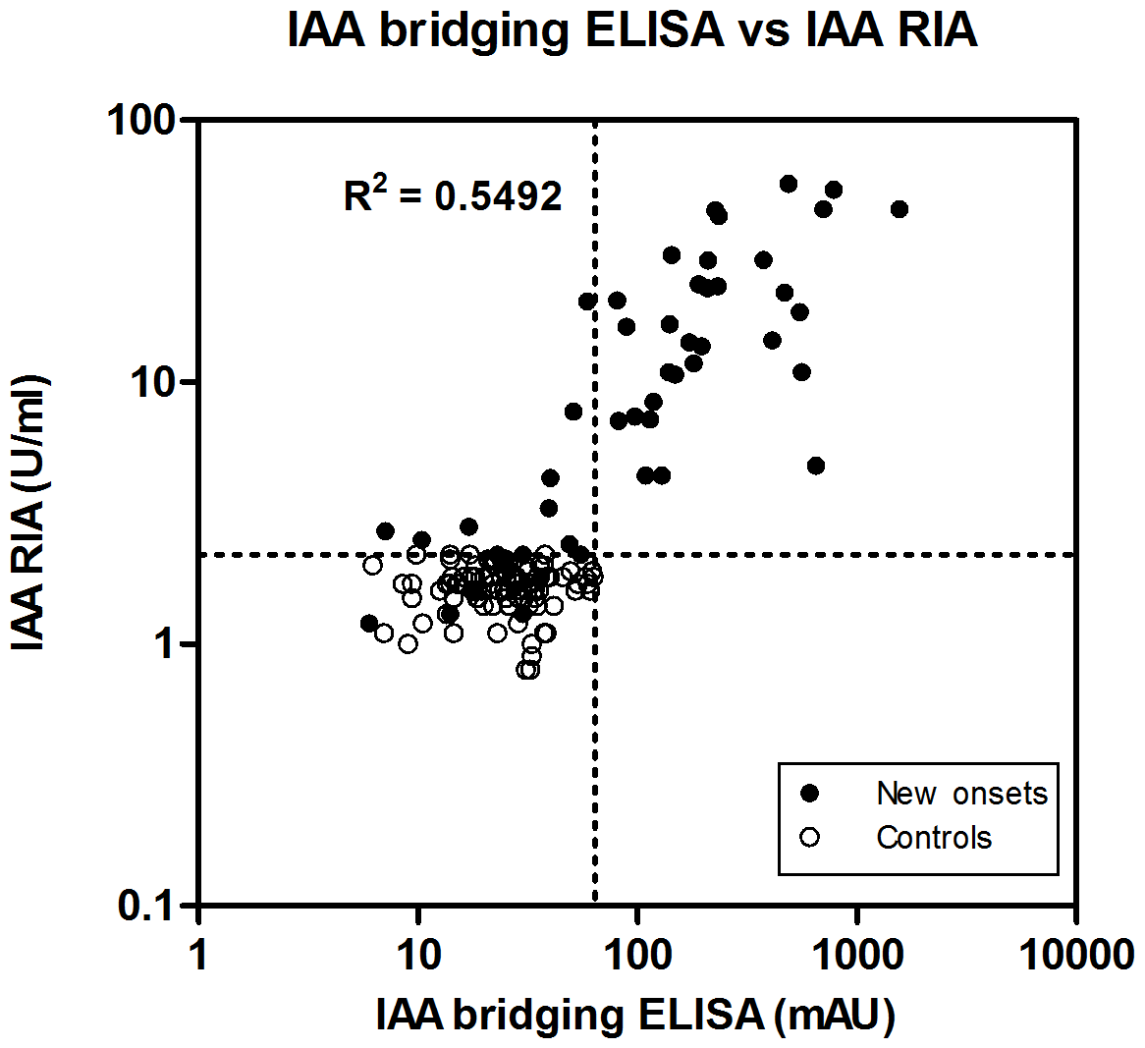
Comparison of IAA levels obtained by IAA bridging ELISA and RIA. Serum samples from 50 newly diagnosed T1D children and 100 control subjects were analyzed with both methods and the two assays were correlated. The dotted lines indicate the cut-off values. mAU, milli-absorbance unit.

**Figure 7:**
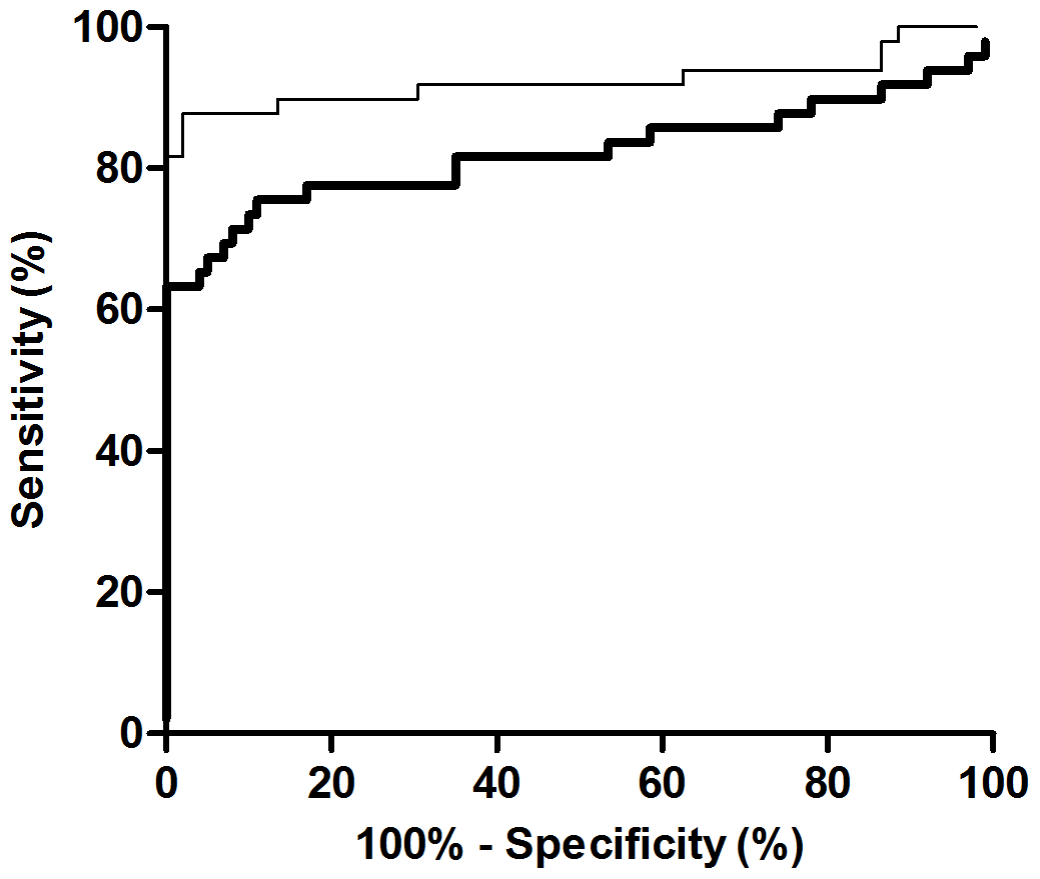
ROC curves for IAA RIA (thin line) and IAA bridging ELISA (thick line). ROC curves result from serum samples from 50 newly diagnosed T1D children and 100 age-matched control children. For IAA RIA, the AUC was 0.92 (95% CI 0.86–0.99) and for IAA bridging ELISA, the AUC was 0.82 (95% CI 0.73–0.91).

The positivity and titers of our IAA bridging ELISA were compared with an IAA RIA (RSR). With IAA RIA, 41 out of 50 (82%) children with newly diagnosed T1D were scored as positive. The results obtained with both assays were correlated using regression analysis (R^2^ = 0.5492; P<0.001; Figure 6). In addition, receiver operator characteristic (ROC) curves for both IAA RIA and IAA bridging ELISA were drawn resulting from serum samples from 50 children with newly diagnosed T1D and 100 control children (Figure 7). For IAA RIA, the area under the curve (AUC) was 0.92 (95% CI 0.86–0.99) and a cut-off value of 64 mAU corresponded to 99% specificity and 82% sensitivity. For IAA bridging ELISA, the AUC was 0.82 (95% CI 0.73–0.91) and a cut-off value of 64 mAU corresponded to 99% specificity and 64% sensitivity. Out of the 9 samples that were IAA RIA-positive but IAA bridging ELISA-negative, 6 subjects were also positive for 2 other islet autoantibodies (IA-2A, GADA) and 3 subjects for one other islet autoantibody (2 for IA-2A and 1 for GADA). These 9 samples resulted in very weak signals with RIA, and 6 of them were barely above the cut-off limit of the assay. We cannot exclude the possibility that conjugating insulin molecules with GC300 and biotin haptens could potentially inhibit the binding of autoantibodies to the antigen. This could possibly explain why these 9 samples were detected by the IAA RIA kit but not with the bridging ELISA. In addition, this discrepancy could be explained by assuming that some paratopes of IAAs are already occupied by circulating free insulin while two free paratopes are required for the bridging assay.

### Conclusion

For assays detecting autoantibodies against GAD and IA-2, results between laboratories are highly concordant [7] and there exists a World Health Organization serum standard for comparing assays in different laboratories and workshops [18]. In contrast, there is poor agreement between laboratories for IAA assays [2].

We describe herein the development of a non-radioactive IAA assay using a bridging ELISA format, which could be a valid alternative to RIAs routinely used in most clinical laboratories. Using our IAA bridging ELISA, IAAs were detected in 32 out of 50 T1D children compared with an IAA RIA scoring 41 out of 50 T1D children as positive. In addition, our IAA bridging ELISA was also compared with an IAA ECL assay carried out using MSD technology. It was found that the limit of detection was very similar for both techniques. Our bridging ELISA has two key advantages. First, no radioactive tracers are required as compared with IAA RIAs. Second, most laboratories are already equipped and trained to perform ELISA-based assays, which is not the case for the MSD technology, which requires specialized and costly equipment. This means that our IAA bridging ELISA could be easily implemented in most clinical laboratories without any special requirements and without the need to pre-treat samples. Moreover, it could be easily adapted to an automated platform. The other key advantage of our bridging assay is its fast readout (8 h).

In summary, our IAA bridging ELISA could be an attractive approach for rapid and automated detection of IAAs in T1D patients for diagnostic purposes. Further validation in at-risk subjects is needed to define its prognostic value for subsequent T1D development.
